# Annexin A1 levels affect microbiota in health and DSS-induced colitis/inflammatory bowel disease development

**DOI:** 10.3389/fimmu.2025.1679071

**Published:** 2025-10-03

**Authors:** Luana Filippi Xavier, Ranko Gacesa, Gustavo Henrique Oliveira da Rocha, Milena Fronza Broering, Pablo Scharf, Fabiana da Silva Lima, Klaas Nico Faber, Hermie Harmsen, Christian Hoffmann, Sandra Helena Poliselli Farsky

**Affiliations:** ^1^ Department of Clinical and Toxicological Analyses, School of Pharmaceutical Sciences, University of São Paulo, São Paulo, Brazil; ^2^ Department of Gastroenterology and Hepatology, University Medical Center Groningen, Groningen, Netherlands; ^3^ Department of Genetics, University of Groningen, University Medical Centre Groningen, Groningen, Netherlands; ^4^ Department of Preclinical Development and Validation, Fraunhofer Institute for Cell Therapy and Immunology, Leipzig, Germany; ^5^ Department of Food and Experimental Nutrition, School of Pharmaceutical Sciences, University of São Paulo, São Paulo, Brazil; ^6^ Department of Medical Microbiology, University Medical Center Groningen, Groningen, Netherlands

**Keywords:** ANXA1, microbiota, DSS-induced colitis, Crohn’s disease, ulcerative colitis, 16S, metagenomics

## Abstract

**Background:**

Inflammatory Bowel Diseases (IBDs) are characterized by intestinal dysbiosis and immune dysregulation. Annexin A1 (AnxA1) promotes epithelial repair and inhibits immune responses during IBD. However, AnxA1’s impact on gut microbiota during IBD remains unclear. Here, we experimentally investigated the microbiota profile during colitis in wild-type (WT) and AnxA1-deficient mice (AnxA1^-/-^), and evaluated an observational cohort in IBD patients with high or low AnxA1 expression.

**Methods:**

Colitis was induced in C57BL/6 WT and AnxA1^^-^/^-^^ mice via oral administration of 2% DSS for six days. Fecal samples were collected at baseline, peak inflammation (day 6), and during the recovery phase (day 10) for 16S rRNA sequencing. Human microbiota data from the Lifelines Dutch Microbiome Project cohort, including IBD and healthy subjects, were analyzed for AnxA1 expression using R software.

**Results:**

Healthy AnxA1^-/-^ mice exhibited reduced microbial richness and a distinct gut microbiota composition, marked by increased *Proteobacteria* and *Parasutterella*, and reduced *Deferribacterota*, *Campylobacterota*, and *Verrucomicrobiota.* During DSS-induced colitis, AnxA1^-/-^ mice showed greater weight loss and heightened inflammation, displaying earlier and more pronounced microbial shifts, including increased *Proteobacteria*, *Cyanobacteria*, *Parabacteroides*, *Bacteroides*, and *Escherichia-Shigella*. In contrast, WT mice exhibited delayed changes, with expansion of *Alloprevotella*, *Akkermansia*, and *Faecalibaculum* after day 6. In human IBD samples, Crohn’s disease (CD) patients with low AnxA1 expression and active inflammation presented an altered microbiota enriched in *Lachnoclostridium* and *Parabacteroides*, while ulcerative colitis (UC) patients showed phylum-level shifts modulated by AnxA1 levels. Notably, non-inflamed CD and UC patients with low AnxA1 differed significantly in microbiota composition. Moreover, inflamed CD patients with high AnxA1 expression showed microbial profiles resembling those of healthy controls, while low AnxA1 expression was associated with a more pronounced dysbiotic state.

**Conclusion:**

AnxA1 is implicated in microbiota control under healthy and IBD conditions. Accordingly, the microbiota of healthy AnxA1^-/-^ mice, colitic AnxA1^-/-^ mice, and IBD patients with low AnxA1 expression exhibit dysbiosis compared to their respective controls. Together, these unprecedented findings reveal AnxA1 as a potential regulatory protein in the immune–microbiota axis involved in IBD pathogenesis.

## 1 Introduction

Inflammatory Bowel Diseases (IBDs), encompassing ulcerative colitis (UC) and Crohn’s disease (CD), are complex disorders influenced by genetic, environmental, and microbial factors. Clinically, IBD symptoms often include diarrhea, abdominal pain, rectal bleeding, and weight loss, with an increased risk of colorectal cancer (1–6). Diagnosis and monitoring rely on invasive procedures such as endoscopy and biopsy, underscoring the need for non-invasive biomarkers to enhance clinical management (7, 8). Treatments range from lifestyle changes and conventional anti-inflammatory drugs to advanced TNF-α, IL-12/IL-23, JAK and integrin inhibitors, as well as surgical procedures (9). However, many patients still experience non-responsiveness or relapse, reinforcing the need for continued research into the multifaceted mechanisms underlying IBDs (10, 11).

Alterations in mucosal integrity and gut microbial composition play a critical role in the progression of IBDs. Patients with IBD exhibit a state of dysbiosis, primarily characterized by reduced microbial diversity and depletion of beneficial phyla such as *Firmicutes* and *Bacteroidetes*, along with an expansion of potentially pathogenic phyla like *Proteobacteria* and *Actinobacteriota* (12, 13). Notably, commensal butyrate-producing bacteria that support mucosal homeostasis and exert anti-inflammatory effects, such as *Faecalibacterium prausnitzii* and *Roseburia* spp. are significantly diminished. In contrast, the abundance of pathobionts, including *Escherichia coli* (adherent-invasive strains), was markedly increased (14–17). This microbial imbalance contributes to a reduction in the production of short-chain fatty acids (SCFAs), particularly butyrate (18), which are essential for maintaining microbial diversity, epithelial integrity, and host–microbiota metabolic balance (19).

Annexin A1 (AnxA1) is a 37 kDa endogenous anti inflammatory protein expressed by neutrophils, monocytes, macrophages, epithelial and cancer cells (20–22). AnxA1 synthesis and release are induced by glucocorticoids and inflammatory mediators. It is found in the cytosol, associated with the plasma membrane or endosomal vesicles, and released via exocytosis or through microvesicles and exosomes (23, 24). Under stress or disease conditions, intracellular AnxA1 can translocate to the nucleus, potentially influencing gene regulation, and also interact with the cytoskeleton, modulating actin and microtubule reorganization (25–28). Secreted AnxA1 undergoes calcium-dependent conformational changes and phosphorylation, exposing its N-terminal domain responsible for its biological activities. Once phosphorylated, AnxA1 binds to membrane phospholipids and to formyl peptide receptors (FPRs), members of the G protein-coupled receptors, and controls inflammation and wound healing (24, 29, 30). Its functions include inhibition of phospholipase A2, neutrophil surveillance, inhibition of leukocyte trafficking into tissues, secretion of chemical mediators, induction of macrophage polarization and efferocytosis and epithelial cell proliferation, adhesion and migration during tissue repair (21, 31, 32). The AnxA1/FPRs’ roles in the development and resolution of inflammation and tissue repair have been extensively studied in IBD experimental and clinical research. In experimental models, AnxA1-deficient mice exhibit exacerbated and non-resolving colitis. In human studies, AnxA1 is highly expressed in inflamed intestinal tissue of IBD patients (21, 33–35).

Understanding AnxA1’s multifunctionality has driven therapeutic strategies, with recent studies indicating that targeting the AnxA1/FPRs pathways may reduce inflammatory disease severity and aid tissue regeneration. Indeed, genetic deficiency of AnxA1 impaired the therapeutic effects of the monoclonal antibody anti-TNF-α or pioglitazone in mice. In contrast, clinical observations in Crohn’s disease patients revealed that those treated with anti-TNF-α who experienced relapse showed lower expression of AnxA1 and FPRs in the inflamed epithelium (33, 36, 37). Emerging nanoparticle-based therapies using AnxA1-mimetic peptides are also being explored to deliver drugs into the inflamed zone, stabilize the intestinal barrier, and promote tissue repair, offering new perspectives in personalized IBD treatment (38–41).

Although the role of AnxA1 in IBD is well recognized, no studies to date have characterized the microbiota profile in the absence of AnxA1, nor the microbial dynamics during IBD development under AnxA1-deficient conditions. To address this, we analyzed the microbiota of wild-type (WT) or AnxA1^-/-^ mice in a DSS-induced colitis model and evaluated a cohort of human IBD patients to identify key differences in microbiota profiles associated with the absence or impaired function of AnxA1.

## 2 Materials and methods

### 2.1 Animals

Male C57BL/6NCrl wild-type (WT) and Annexin A1-deficient (AnxA1^-/-^) mice aged 7–8 weeks and weighing 20–25 g were obtained from the Animal Facility of the School of Medicine at UNIFESP. Mice had conventional sanitary standards and were kept in barrier conditions on a 12-hour light/dark cycle at temperatures between 20 and 25°C with free access to water and food. All procedures were performed following the Ethical Principles for Animal Experimentation by the National Council for Animal Experimentation Control (CONCEA). The protocol of the study was approved by the Ethics Committee of Animal Use of the Faculty of Pharmaceutical Sciences of the University of São Paulo (CEUA/FCF/USP, protocol n° 617) and the Internal Biosafety Commission (CIBio).

### 2.2 Experimental colitis induced by dextran sulfate sodium

To induce experimental colitis, 2% dextran sulfate sodium (DSS; MW 40,000, Dextran Products Limited, Ontario, Canada) was administered in the drinking water for six consecutive days (days 0–6), while control animals received only filtered water. Wild-type (WT) and AnxA1^-/-^ mice were divided into two groups: DSS-treated and control, and the experimental protocol lasted 10 days in total (**Supplementary Figure 1A**).

### 2.3 Assessment of colitis clinical score and FACS

Colitis development was monitored daily by body weight measures, in which the progression was expressed as the difference between initial and final weight, as percentage. On day 10 of the experiment, animals were anesthetized with ketamine (90 mg/kg) and xylazine (10 mg/kg) via intraperitoneal injection. A surgical incision was made in the mice’s abdominal region and the colon was removed from the ileocecal junction to the anus, washed with an isotonic solution and the organ was fragmented for FACS analysis. Intestinal tissue was subjected to enzymatic digestion with collagenase, filtered through a 40 μm strainer, and washed with PBS. After centrifugation, cells were resuspended in FACS buffer (PBS, 5% FBS, 0.02% NaN_2_) and incubated on ice for 15 min to block nonspecific binding (42). Cells were stained with anti-Ly6G (1:50, BD Biosciences), anti-F4/80 (1:100, eBioscience), anti-CD3e (1:50, eBioscience) and anti-CD4 (1:100, Biolegend) antibodies. Analyses were performed on a BD Accuri C6 Plus cytometer and analyzed using BD Accuri C6 software.

### 2.4 Experimental statistical analyses

All results were subjected to normality tests, including D’Agostino-Pearson and Kolmogorov-Smirnov. Clinical analyses were evaluated using Two-way ANOVA, followed by Tukey’s post-test, with results expressed as mean ± standard deviation. Tissue analyses were performed using One-way ANOVA with Tukey’s post-test and expressed as mean ± standard deviation. Values of p<0.05 were considered significant. Statistical analyses were performed using GraphPad Prism version 8.0 (GraphPad Software, San Diego, CA, USA).

### 2.5 DNA sequencing of fecal mice samples

Feces were harvested on days 0, 6 and 10 of the experiment for 16S sequencing of the intestinal microbiota. DNA was extracted from the feces using the DNeasy Power Soil Kit (Qiagen) and Power Soil Pro Kit (Qiagen). The V4 region of the 16S ribosomal gene was amplified using the AccuPrime™ Taq DNA Polymerase System (Invitrogen), using the primers 515F–806R, as described by Caporaso et al. (43) and protocolized in the Earth Microbiome Project. Genetic material was purified using beads via the Beckman Coulter Agencourt AMPure XP Kit (Beckman Coulter), and quality and size were checked by gel electrophoresis. DNA quantification was performed using the Quant-iT PicoGreen dsDNA Kit (Invitrogen). Sequencing was performed on the Illumina MiSeq^®^ System (Illumina INC, San Diego, CA, USA) at the Research Support Facilities Center (CEFAP) - ICB-USP, following the manufacturer’s protocol, using the Illumina V2 500-cycle kit (Illumina).

### 2.6 Experimental bioinformatics analyses

Bioinformatics analyses were performed using the QIIME2 software version 2022.2. Sequences were imported into the program and demultiplexed to associate indexes with their respective samples. A total of 5,011,692 sequences were obtained, with an average of 27,998 sequences per sample. The sequences were processed through the Dada2 pipeline to filter low-quality sequences, singletons, chimeric sequences, and to dereplicate sequences to reduce repetition. The first 10 bases of the sequence were trimmed, and sequences were truncated at base 248 based on data quality scores. Results were tables of Amplicon Sequence Variants (ASVs), containing information about the frequency and sequence of ASVs in the samples. After quality control with Dada2, 3,888,180 sequences were obtained across all samples, resulting in 1,574 ASVs. Diversity and taxonomy analyses were then performed, using a sampling depth of 10,367. The ASVs were aligned using the mafft pipeline and used to build phylogeny with fast tree. Alpha diversity was assessed using Faith and Evenness metrics with statistical analysis by Kruskal-Wallis, while beta diversity was analyzed using UniFrac weighted and unweighted, with PERMANOVA and principal coordinate analysis (PCoA). Taxonomy analysis was performed using the q2‐feature‐classifier pipeline with Silva database (silva-138-99-nb-weighted-classifier). All analyses were performed using rarefied tables subsampled to 10,367 sequences per sample.

### 2.7 Patient cohort and ethics committee

The data used for bioinformatics analyses in humans originates from the Lifelines Dutch Microbiome Project (DMP) cohort, a multidisciplinary prospective study designed to monitor health and health-related behaviors in 167,729 individuals living in the northern Netherlands. For this study, data from a total of 882 individuals were analyzed, including 458 individuals with Crohn’s disease, 363 with ulcerative colitis, and 61 controls. The IBD patients were further subdivided based on the presence or absence of biopsy tissue inflammation and the expression level of the AnxA1 protein (high vs. normal-low). The Lifelines study was approved by the ethics committee of the University of Groningen under the protocol number METc: 2017/152. The data can be accessed and/or requested through the following website: https://ega-archive.org/studies/EGAS00001005027.

### 2.8 Human cohort bioinformatics analyses

The bioinformatics analyses for the translational component were performed using the R software, version 4.3.1. Pre-normalized text files containing sample counts were provided for use in the study. For alpha diversity analysis, a minimum filter of 50 counts per genus was applied. The Vegan package was used to calculate Shannon, Richness, Evenness, and Chao1 metrics. Normality was tested using the Shapiro-Wilk test, and statistical differences were analyzed using the Kruskal-Wallis test followed by Dunn’s *post hoc* test. For beta diversity analysis, a filter of 50 counts per genus was applied, and the relative abundance of each genus within each sample was calculated. Using the Vegan package, Bray-Curtis distance was computed, and PERMANOVA was used for statistical analyses, with pairwise comparisons conducted using the Pairwise package. For the taxonomic analysis, the data were transformed into relative abundance and visualized using the ggplot2 package.

## 3 Results

### 3.1 The deficiency of AnxA1 influences the gut microbiota of healthy mice

**Figure 1:**
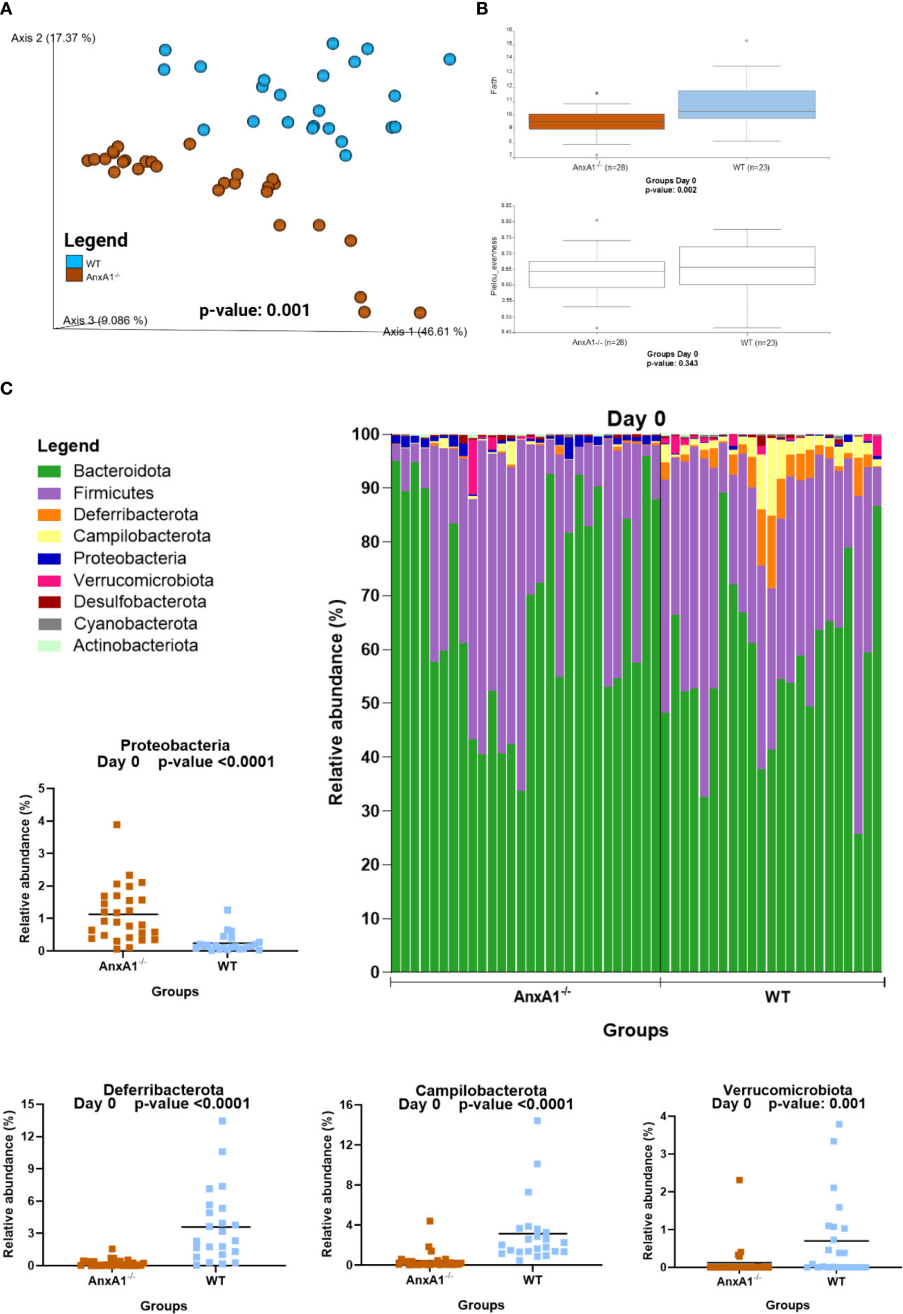
Microbiota shifts in the absence of AnxA1 in basal conditions (Day 0). **(A)** UniFrac weighted metric for beta diversity comparing WT and AnxA1^-/-^ mice. P-value: 0.001 **(B)** Alpha diversity comparing WT and AnxA1^-/-^ mice performing Richness (upper graph; p-value: 0.002) and Evenness (bottom graph; p-value: 0.343) metrics. **(C)** Relative abundance of phyla between WT and AnxA1^-/-^ mice. ASVs tested with Mann-Whitney. *Proteobacteria, Deferribacterota* and *Campilobacterota* p-values <0.001, while *Verrucomicrobiota*: 0.001. *n*= WT: 23 and AnxA1^-/-^: 28. For all analyses, p-values <0.05 were considered statistically significant.

Healthy AnxA1^-/-^ mice exhibited a distinct gut microbiota profile compared to the WT mice. Beta diversity analysis at day 0 revealed a significant difference between groups (**Figure 1A**). Regarding alpha diversity, AnxA1^-/-^ mice showed reduced ASV richness, while evenness did not differ between groups (**Figure 1B**).

**Figure 2:**
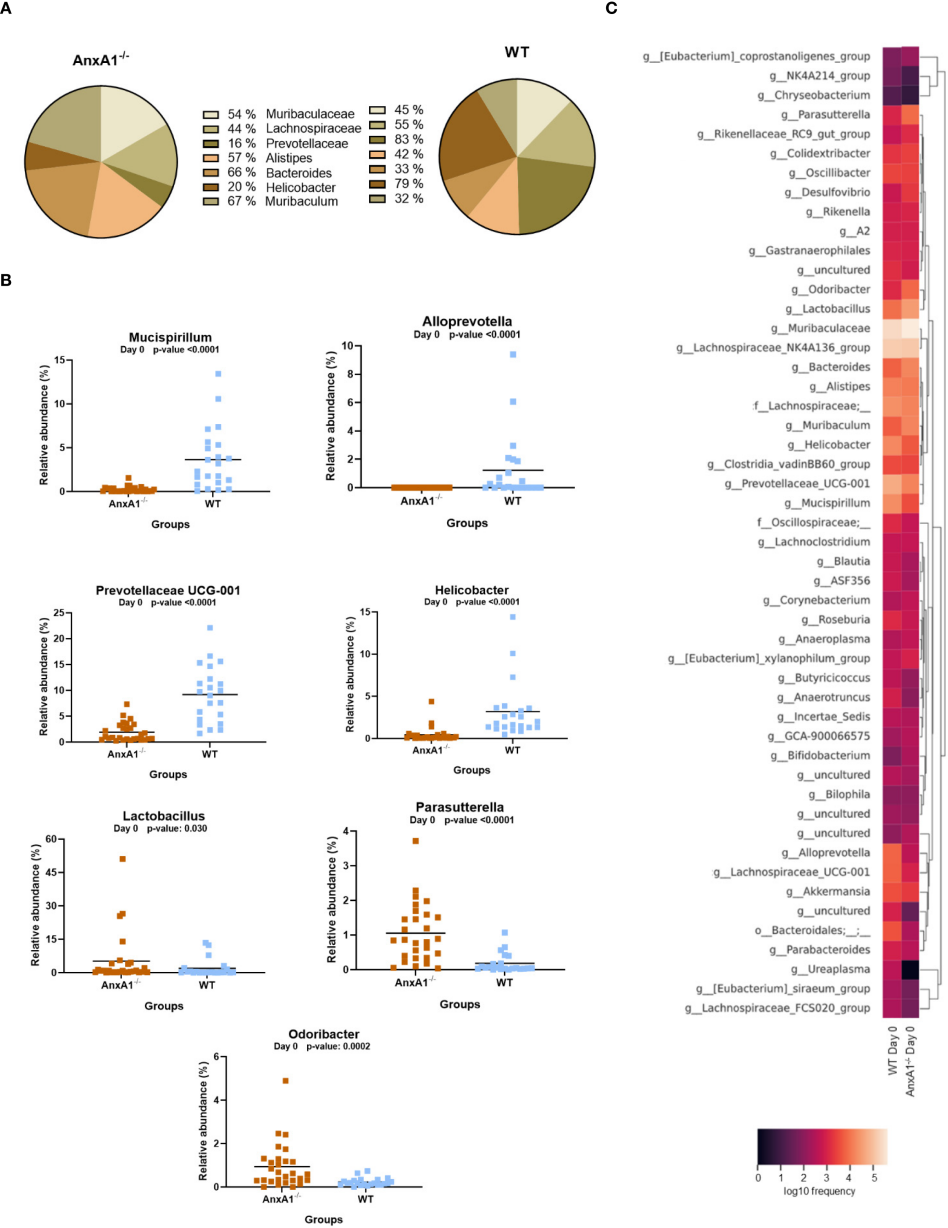
Genera shifts in the absence of AnxA1 in basal conditions (Day 0). **(A)** Percentage graph of the main genera commonly found in WT and AnxA1^-/-^ mice. **(B)** Relative abundance of genera between WT and AnxA1^-/-^ mice. ASVs tested with Mann-Whitney. *Mucispirillum, Alloprevotella, Prevotellaceae UCG-001, Helicobacter* and *Parasutterella* p-values<0.001. *Lactobacillus* p-value: 0.030 and *Odoribacter*: 0.0002. **(C)** Heatmap with the main shifts in genus level between WT and AnxA1^-/-^ mice. *n*= WT: 23 and AnxA1^-/-^: 28. For all analyses, p-values <0.05 were considered statistically significant.

In AnxA1^-/-^ mice, a higher prevalence of the phylum *Proteobacteria* was observed, while *Deferribacterota*, *Campylobacterota*, and *Verrucomicrobiota* were reduced compared to the WT group (**Figure 1C**). Within these phyla, AnxA1^-/-^ mice exhibited higher abundance of *Parasutterella* (a member of *Proteobacteria*), along with decreased abundance in *Helicobacter* (a member of *Campylobacterota*) and *Mucispirillum* (a member of *Deferribacterota*) compared to WT mice (**Figure 2B**).

The main genera found in both mouse strains are presented in **Figure 2A**. In the absence of AnxA1, significant differences were also observed in less abundant genera. For instance, *Odoribacter* and *Lactobacillus* were more abundant in AnxA1^-/-^ mice, whereas *Prevotellaceae UCG-001* was less abundant compared to WT. Notably, *Alloprevotella* was detected exclusively in WT mice (**Figure 2B**). The primary differences in genus level in healthy animals are visualized in the heatmap in **Figure 2C**.

FACS analysis of the colon revealed that in healthy conditions, WT and AnxA1^-/-^ mice showed similar percentages of colonic Ly6G^+^ neutrophils, F4/80^+^ macrophages, and CD3^+^ T lymphocytes (**Supplementary Figure 1B**).

### 3.2 Microbiota exhibits pronounced inflammatory profile in AnxA1^-/-^ mice during colitis development

Between days 6 and 10 of DSS-induced colitis, all treated groups showed significant weight loss compared to controls, with AnxA1^-/-^ DSS mice exhibiting greater loss on days 6 to 8 (**Supplementary Figure 1C**), suggesting a more severe inflammatory response in the absence of AnxA1, consistent with previous studies (33, 36, 37). During colitis, AnxA1^-/-^ mice exhibited a marked increase in Ly6G^+^ neutrophils and reduced influx of F4/80^+^ macrophages compared to WT. The percentage of CD3^+^ T cells declined in AnxA1^-/-^ mice during inflammation, while remaining stable in WT. Interestingly, CD4^+^ T cells were more abundant in healthy AnxA1^-/-^ mice, but this population significantly decreased during colitis, whereas it remained unchanged in WT animals (**Supplementary Figure 1B**).

**Figure 3:**
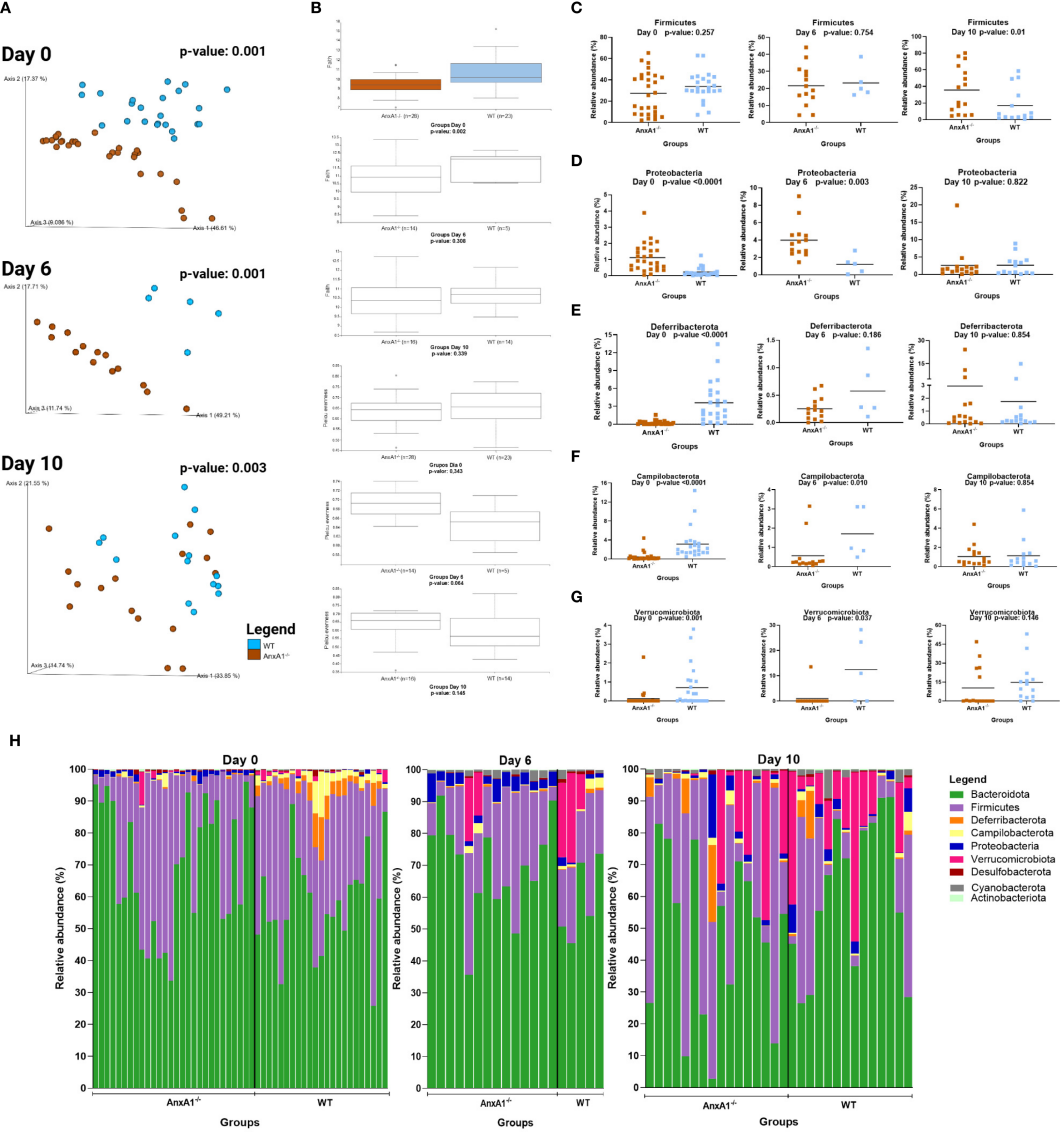
Microbiota shifts during DSS-induced colitis between WT and AnxA1^-/-^ mice. **(A)** UniFrac weighted metric for beta diversity comparing WT and AnxA1^-/-^ mice through the colitis model. P-value day 0 and 6 = 0.001, day 10 = 0.003. **(B)** Alpha diversity comparing WT and AnxA1^-/-^ mice performing Richness (upper graph; p-values day 0 = 0.002, day 6 = 0.308 and day 10 = 0.339) and Evenness (bottom graph; p-values day 0 = 0.343, day 6 = 0.064 and day 10 = 0.145) metrics. **(C-G)** Relative abundance of phyla between WT and AnxA1^-/-^ mice. ASVs were tested with Mann-Whitney. **(H)** Relative abundance shifts for all phyla through the colitis model. *n* day 0 WT = 23 and AnxA1^-/-^= 28; *n* day 6 WT = 5 and AnxA1^-/-^= 14; *n* day 10 WT = 14 and AnxA1^-/-^=16. For all analyses, p-values <0.05 were considered statistically significant.

Day 6 of the model corresponds to the peak of clinical manifestations, while day 10 marks the recovery phase of the disease. As observed under healthy conditions, the bacteria composing the gut microbiota during experimental colitis maintain an individual pattern in the presence or absence of AnxA1 (**Figure 3A**). No differences in richness or evenness of the ASVs were detected between the groups through the colitis (**Figure 3B**).

The most abundant phyla in both groups remained *Bacteroidota* and *Firmicutes* before and after inducing colitis. However, the abundance of *Firmicutes* was increased in AnxA1^-/-^ compared to WT on the 10th day (**Figure 3C**). Although AnxA1^-/-^ maintained higher abundance of *Proteobacteria* in the peak of inflammation, this difference between groups lost significance on day 10 though (**Figure 3D**). *Deferribacterota* and *Campilobacterota* phyla were less abundant in AnxA1^-/-^ mice on day 0, with slight variation on day 6 and 10. In contrast, WT mice lost abundance of this phylum through the development of the disease, so no more differences were observed between the two groups over the days (**Figures 3E, F**). *Verrucomicrobiota* bacteria were increased in both groups with colitis, although lower abundance of these bacteria was observed in AnxA1^-/-^ mice in the beginning, and on the 10^th^, these differences disappeared (**Figure 3G**). The changes in the microbiota phyla profile through colitis can be observed in **Figure 3H**.

**Figure 4:**
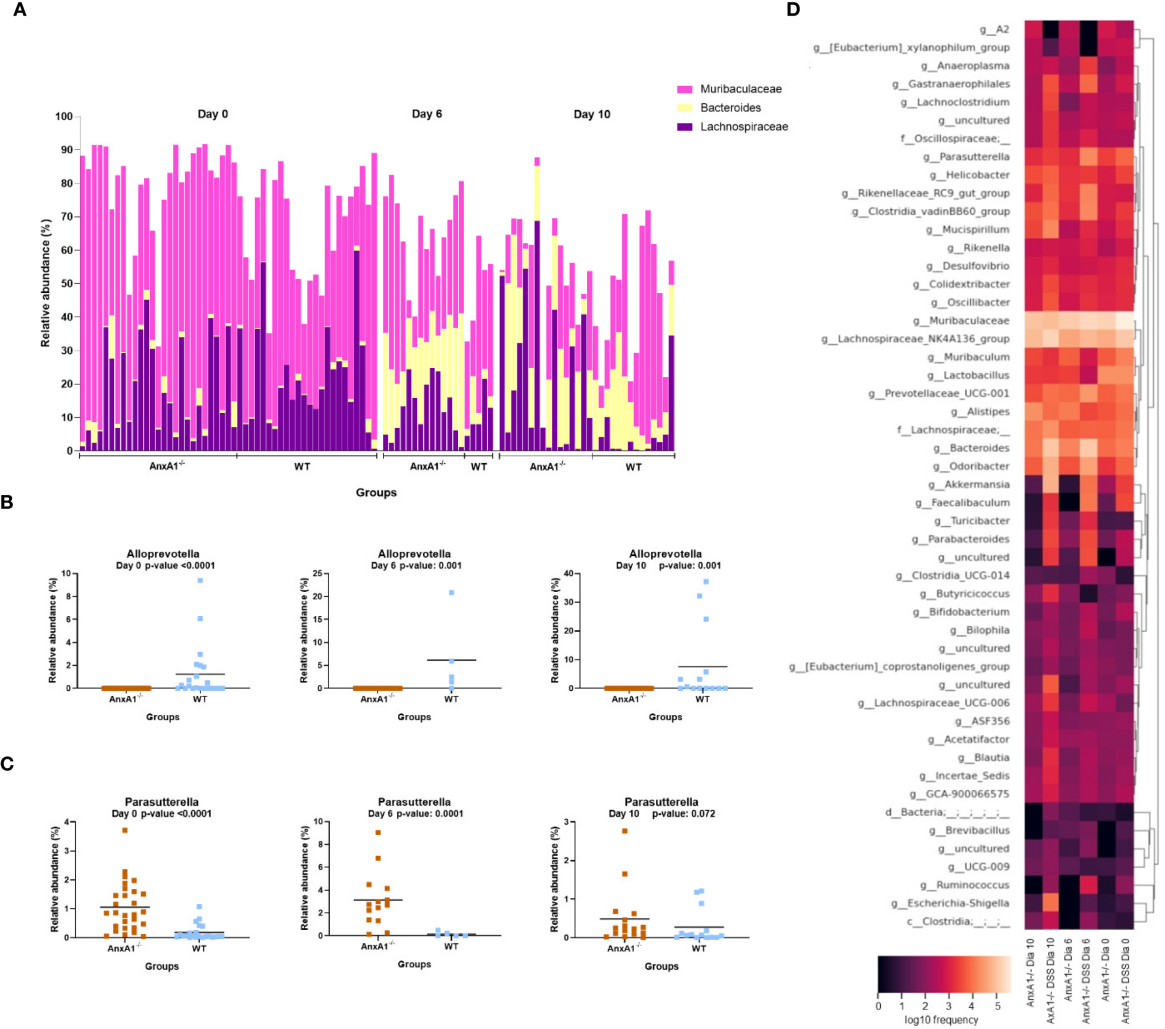
Genera shifts during DSS-induced colitis between WT and AnxA1^-/-^ mice. **(A)** Relative abundance shifts for the genera belonging to *Lachnospiraceae NK4A136, Bacteroides* and *Muribaculaceae* through the colitis model. **(B, C)** Relative abundance shifts for Alloprevotella and *Parasuterella* genera through the colitis model. ASVs were tested with Mann-Whitney. **(D)** Heatmap with the main shifts in genus level between WT and AnxA1^-/-^ mice through the colitis model. *n* day 0 WT = 23 and AnxA1^-/-^= 28; *n* day 6 WT = 5 and AnxA1^-/-^= 14; *n* day 10 WT = 14 and AnxA1^-/-^=16. For all analyses, p-values <0.05 were considered statistically significant.

The genera *Lachnospiraceae* NK4A136 and *Bacteroides* increased on day 10, overlapping the prevalence of *Muribaculaceae* in the AnxA1^-/-^ animals (**Figure 4A**), while *Alloprevotella* continued to be identified only in WT mice (**Figure 4B**). The genus *Parasuterella*, more abundant at the beginning of the disease, increased in abundance on the peak of inflammation and decreased on the 10th day; no differences were observed in WT DSS mice (**Figure 4C**). The shift in abundance at the genus level across the days can be observed in the heatmap in **Figure 4D**.

### 3.3 AnxA1^-/-^ mice are more susceptible to changes in microbiota during experimental colitis

**Figure 5:**
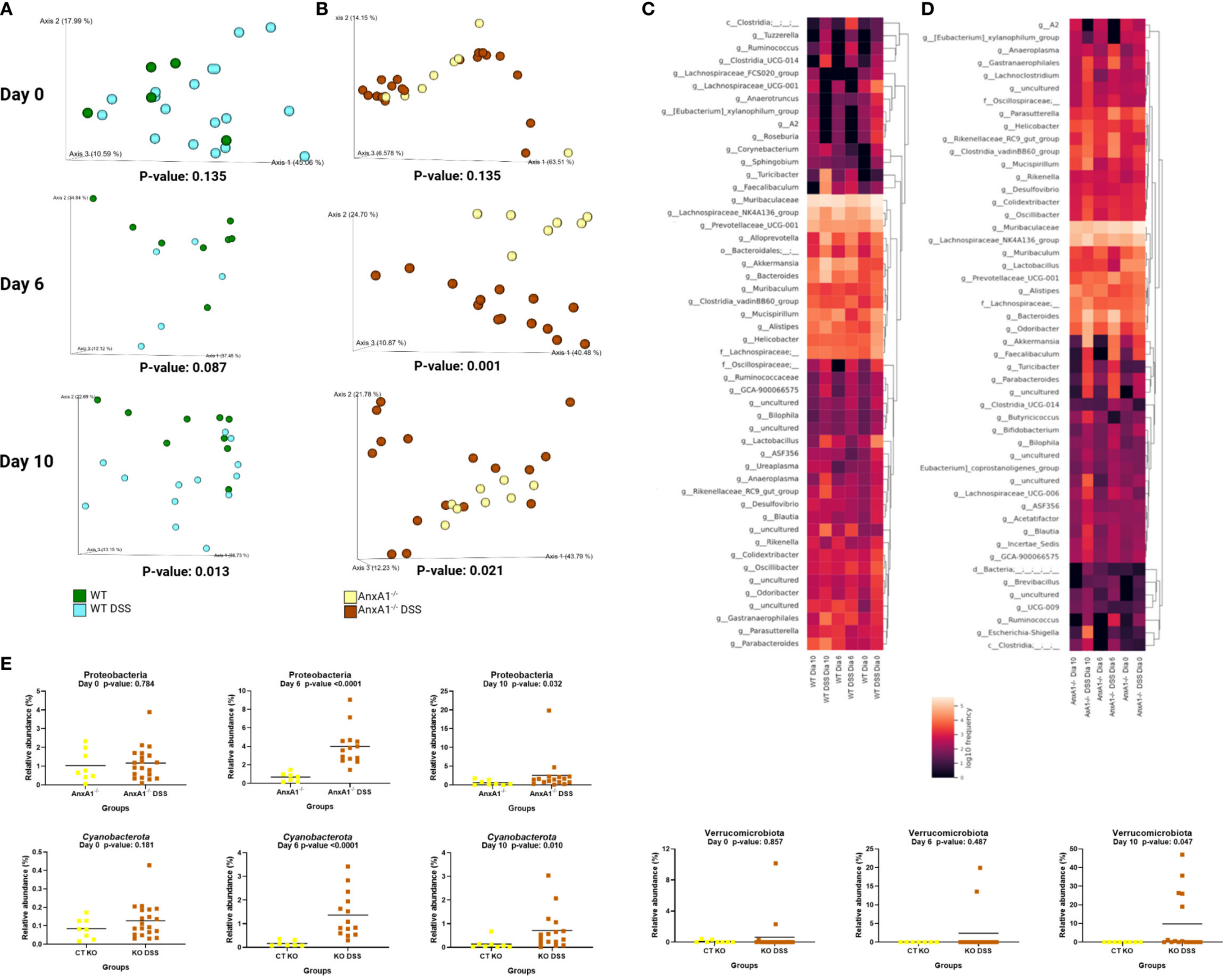
Early alterations in microbiota profile in the absence of AnxA1. **(A)** UniFrac weighted metric for beta diversity comparing WT control and WT DSS mice through the colitis model. P-values day 0 = 0.135, day 6 = 0.087 and day 10 = 0.013. **(B)** UniFrac weighted metric for beta diversity comparing AnxA1^-/-^ control and AnxA1^-/-^ DSS mice through the colitis model. P-values day 0 = 0.135, day 6 = 0.001 and day 10 = 0.021. **(C)** Heatmap with the main shifts in genus level between WT control and WT DSS mice through the colitis model. **(D)** Heatmap with the main shifts in genus level between AnxA1^-/-^ control and AnxA1^-/-^ DSS mice through the colitis model. **(E)** Relative abundance of phyla between AnxA1^-/-^ and AnxA1^-/-^ DSS mice. ASVs were tested with Mann-Whitney. *n* day 0 WT = 5 and WT DSS = 18, AnxA1^-/-^= 8 and AnxA1^-/-^ DSS = 20; *n* day 6 WT = 8 and WT DSS = 5, AnxA1^-/-^= 7 and AnxA1^-/-^ DSS = 14; *n* day 10 WT = 9 and WT DSS = 13, AnxA1^-/-^= 8 and AnxA1^-/-^ DSS = 16. For all analyses, p-values <0.05 were considered statistically significant.

The microbiota of WT mice started to meaningfully change from a healthy condition after the 6^th^ day of the disease, as shown in **Figure 5A**. In contrast, in the absence of AnxA1, differences in microbial diversity were already observed on day 6 (**Figure 5B**).

In WT mice, the most notable shifts were increments in *Turicibacter*, *Alloprevotella*, *Akkermansia*, *Bacteroides*, and *Faecalibaculum* genera. Modifications detected in lesser pronounced genus levels are presented in **Figure 5C**.

In contrast, AnxA1^-/-^ mice exhibited earlier shifts at the phylum level, with increased abundance of *Proteobacteria* and *Cyanobacteria* already detectable on day 6 (**Figure 5E**). On day 10, a notable enrichment of the *Verrucomicrobiota* phylum was observed. However, the most prominent changes at the genus level were associated with members of the *Firmicutes* and *Bacteroidota* phyla. These included increased levels of *Parabacteroides*, *Bacteroides* and *Turicibacter*. *Gastranaerophilales*, a member of the *Cyanobacteriota* phyla and *Escherichia-Shigella* genera from the *Proteobacteriota* phyla were also enriched by the end of the disease in AnxA1^-/-^ mice (**Figure 5D**).

### 3.4 Different expression in Annexin A1 levels affects the microbiota in IBD patients

**Figure 6:**
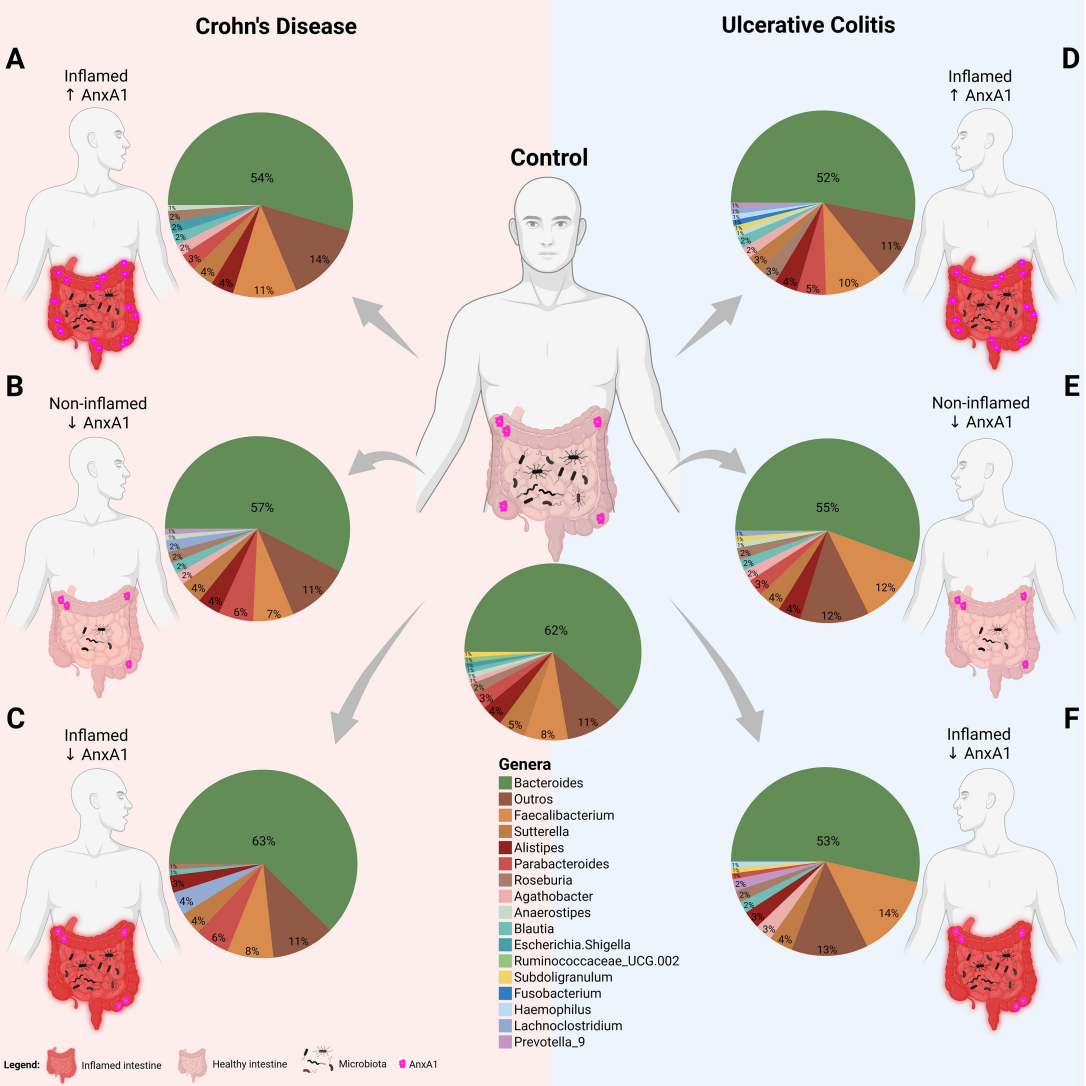
Microbiota shifts in IBD with AnxA1 differential expression. Relative abundance of main bacterial genera shifts in the colon of CD and UC subjects compared to control (centered). **(A)** CD patient with inflamed colon and high AnxA1 expression. **(B)** CD patient non-inflamed with basal AnxA1 expression. **(C)** CD patient with inflamed colon and low levels of AnxA1 expression. **(D)** UC patient with inflamed colon and high AnxA1 expression. **(E)** UC patient non-inflamed with basal AnxA1 expression. **(F)** UC patient with inflamed colon and low levels of AnxA1 expression.

The identified phyla in the human cohort subjects were *Acidobacteriota, Actinobacteriota, Bacteroidota, Cyanobacteria, Campylobacterota*, *Firmicutes, Fusobacteriota, Patescibacteria, Proteobacteria, Spirochaetota*, and *Verrucomicrobiota*. The most abundant bacterial genera found in this study were *Bacteroides, Faecalibacterium, Parabacteroides, Sutterella, and Alistipes* (**Figure 6**).

CD patients with active inflammation and low AnxA1 expression exhibited distinct microbiota composition compared to healthy controls, mainly presenting an increase in *Lachnoclostridium* and *Parabacteroides* genera (**Figure 6C**). This pattern was not observed in inflamed patients with high AnxA1 levels though (**Figure 6A**), suggesting that elevated AnxA1 may partially preserve the microbial profile, maintaining it at an intermediate level, still dysbiotic, but less disrupted than in low levels of AnxA1. Moreover, CD patients in the relapse phase with basal or low AnxA1 expression (**Figure 6B**) showed no significant differences in microbiota composition when compared to healthy controls, but reduced bacterial richness.

UC patients presented the same microbial genera composition regardless of AnxA1 expression level (**Figures 6D-F**). However, in active disease, the magnitude of AnxA1 expression influenced the phylum, class and order levels of bacteria compared to relapsing patients. In this scenario, high levels of AnxA1 reflected in an increase at the *Actinobacteriota* and *Bacteroidota* phyla, as an increase in *Parabacteroides*, although the latter has not reached statistical significance (**Figure 6D**). Likewise, active disease in UC patients with low AnxA1 levels was marked by an increase in *Faecalibacterium* and *Prevotellaceae*, along with a decrease in *Parabacteroides*, although not significantly (**Figure 6F**). This shift reflects a more dysbiotic and pro-inflammatory microbial profile in the absence of AnxA1. Altogether, these findings suggest impaired anti-inflammatory regulation in AnxA1 deficiency.

If the microbiota of both IBD diseases are compared, it is possible to infer that the disease type may influence bacterial composition in the AnxA1-reduced condition. Non-inflamed CD and UC patients with reduced AnxA1 expression displayed different microbiota profiles (p-value <0.01), with higher abundance of *Lachnoclostridium* and *Parabacteroides* in CD patients, and lower abundance of *Faecalibacterium* (**Figures 6B, E**). Bacterial genus richness and evenness were also lower in CD patients. Together, the data obtained here point out that levels of AnxA1, even in the latent or relapsing inflammation, affect the microbiota profile in humans.

## 4 Discussion

The gut microbiota and the immune system are tightly interconnected, and intestinal homeostasis relies on a delicate balance between microbial surveillance, inflammatory responses and their resolution (44). Disruption of this balance, particularly due to deficiencies in pro-resolving mediators, such as AnxA1, impairs the regulation of inflammation and contributes to the pathogenesis of IBDs (33, 37, 41, 45). Although the immunomodulatory roles of AnxA1 on IBD have been described, its influence on microbial dynamics remains poorly understood. In this study, unprecedented data strengthen the evidence for AnxA1’s involvement in microbiota modulation, either dependent or independent of the inflammatory state.

Healthy AnxA1-deficient mice presented reduced richness in comparison to WT, which, along with compositional differences in the bacterial community, suggests resilient or functionally limited microbiota with predisposition to exaggerated inflammation upon stimulation. Among the taxonomic shifts, AnxA1^-/-^ mice presented the overgrowth of *Proteobacteria*, which is considered a marker of microbiota instability, leading to immune activation, associated with Toll-like receptor (TLR) expression and inflammatory cytokines release, such as TNF-α, IL-6, and IL-1β (46), rendering the host more susceptible to inflammatory stimuli (18, 47–49). Notwithstanding, a reduction in microbial richness, as observed in AnxA1^-/-^ mice, has been correlated with a relative expansion of *Proteobacteria* in IBDs (50). Consistent with this pattern, an increase in *Parasutterella*, which is linked to metabolic and inflammatory imbalances (51), indicates underlying alterations in epithelial barrier integrity or immune signaling pathways in the absence of AnxA1. In addition, the lower abundance of *Deferribacterota* and *Campylobacterota* in healthy AnxA1^-/-^ mice, including their representative genera *Mucispirillum* and *Helicobacter*, which typically colonize mucus-adjacent niches, suggests that AnxA1 influences mucosal microbial colonization under physiological conditions. Notably, *Helicobacter hepaticus*, a species within *Campylobacterota*, has been implicated in shaping Th17 responses, being considered a pathobiont strain by disrupting Treg/Th17 axis in immunodeficient conditions (52).

*Helicobacter* was more abundant in WT and AnxA1^-/-^ mice under basal conditions, but its levels markedly increased in the absence of AnxA1 during colitis, erasing the initial differences. This expansion under inflammatory stress may reflect the inability of AnxA1^-/-^ mice to effectively restrain immune activation, allowing overgrowth of potentially pro-inflammatory taxa. While *Helicobacter*-driven IL-17A responses (52) may be tightly regulated in WT mice, maintaining mucosal balance, its dysregulation in AnxA1^-/-^ animals could amplify inflammation and worsen disease outcomes.

Another genus decreased in AnxA1^-/-^ mice was *Prevotellaceae UCG-001*, a bacterium associated with fiber fermentation and SCFA production (53). Its reduction may reflect impaired microbial metabolic function and a shift toward a more pro-inflammatory gut environment (54). The absence of *Alloprevotella* in AnxA1^-/-^ mice is also notable, given that this genus is typically associated with the production of SCFAs. Moreover, its presence is often linked to greater microbial diversity, which is generally considered favorable for gut health (55, 56). In contrast, the higher abundance of *Lactobacillus* and *Odoribacter* in AnxA1^-/-^ mice may reflect a microbial response associated with the maintenance of intestinal homeostasis. *Odoribacter*, for instance, can modulate macrophage function and epithelial barrier integrity through its metabolites. Loss of *Odoribacter* in colitis is linked to reduced SCFA availability, leading to intestinal inflammation (57, 58). Meanwhile, in dysbiosis, *Lactobacillus* may promote Th1 responses, enhancing the secretion of inflammatory cytokines like IFN-γ and TNF-α, contributing to IBD development (59). However, the functional impact of this shift remains unclear, as quantitative changes do not always translate into equivalent functional outcomes. The associated data clearly show a basal difference in microbiota constitution of AnxA1^-/-^, clearly showing that alteration in genotype affected bacteria in the gut. Although the leukocyte population in the gut of healthy AnxA1 or WT are similar, it remains to be evaluated how the absence of AnxA1 affects the microbiota.

As observed in the healthy state, the microbiota of AnxA1^-/-^ and WT mice maintained distinct profiles throughout the colitis timeline, suggesting that the lack of AnxA1 actively drives microbiota divergence even under inflammatory conditions. The persistent enrichment of *Proteobacteria* in AnxA1^-/-^ mice during the peak of inflammation (day 6) is consistent with prior reports linking this phylum to intestinal dysbiosis, potentially contributing to a more severe inflammatory profile compared to the WT group (60). Furthermore, dysbiosis with a predominance of *Proteobacteria* may lead to persistent innate immune activation, intensifying neutrophil and macrophage infiltration into the intestinal mucosa and worsening chronic inflammation (61). Interestingly, by day 10, this difference was no longer significant between both genotypes, which may reflect a delayed or altered resolution of the inflammatory response in the absence of AnxA1.

*Firmicutes*, one of the dominant phyla in the healthy gut, were more abundant in AnxA1^-/-^ DSS mice at day 10, potentially indicating a compensatory bloom of taxa involved in butyrate production during the recovery phase (62). However, the functional relevance of this expansion remains to be elucidated. The progressive increase in *Verrucomicrobiota* across colitis is aligned with reports of *Akkermansia muciniphila* expansion during epithelial barrier damage or remodeling (63). Also, it was observed that the enrichment of *Akkermansia* through the days in both groups of animals. The disappearance of initial differences by day 10 between the groups may indicate a common mucosal healing process in both genotypes. Importantly, the persistence of *Alloprevotella* exclusively in WT mice across time points reinforces its sensitivity to the AnxA1-dependent mucosal environment. Given the association of this genus with SCFA production and immunoregulatory effects, its absence in AnxA1^-/-^ mice may contribute to their distinct inflammatory profile.

Additionally, the increase of *Lachnospiraceae NK4A136* group and *Bacteroides* in AnxA1^-/-^ mice by day 10, overtaking *Muribaculaceae*, may reflect a shift in fermentative capacity or epithelial nutrient availability during recovery (64, 65). *Bacteroides* enrichment during recovery may not signal resolution, but rather a dysbiotic rebound shaped by host-microbiota disequilibrium (66–68). *Parasutterella*, initially enriched in AnxA1^-/-^ mice, showed a transient bloom at peak inflammation before returning to WT levels, which may indicate a context-dependent interaction with host immunity or metabolic state. These bacteria produce succinate, which aids microbiota recovery but also promotes ulceration in DSS-colitis (69). In IBDs, excessive succinate drives inflammation by activating immune cells, HIF-1α, and IL-1β, while also fueling pathogens like *C. difficile*, disrupting microbial balance (70–72).

The dynamics of gut microbiota during experimental colitis reveal a distinct temporal pattern between WT and AnxA1^-/-^ mice, suggesting that AnxA1 plays a critical role in modulating the microbial response to inflammation. In WT mice, meaningful microbiota changes only emerged later during disease progression in comparison to AnxA1^-/-^, whereas AnxA1^-/-^ mice exhibited earlier shifts. These findings suggest that the absence of AnxA1 predisposes the gut environment to dysbiosis more rapidly during inflammatory stress, in line with its known regulatory functions in mucosal immunity and tissue homeostasis (73).

Although both WT and AnxA1^-/-^ mice developed colitis, the heightened severity of the model may have obscured subtle differences between strains. Nevertheless, AnxA1^-/-^ mice showed greater weight loss and increased circulating neutrophils, suggesting a more intense and unresolved inflammatory response. This is consistent with the known role of AnxA1 in regulating neutrophil physiology, including their release from the bone marrow, migration to inflamed tissues, apoptosis, and clearance by M2 macrophages in homing tissues (24, 74–76). In its absence, these processes become dysregulated, leading to persistent neutrophilia and impaired resolution. Additionally, the observed lymphopenia likely reflects lymphocyte recruitment to the inflamed colon, a process also influenced by AnxA1 through its modulation of leukocyte trafficking (34, 76, 77).

While AnxA1 also governs monocyte infiltration and promotes anti-inflammatory M2 macrophage polarization, no increase in macrophage numbers was detected by day 10 of colitis. This may be due to a predominance of pro-inflammatory M1 macrophages in AnxA1^-/-^ mice or limitations in detection, as F4/80 alone may not adequately distinguish between macrophage subtypes, highlighting the need for more specific markers in future analyses (26, 78–80).

Together, these data indicate that AnxA1 plays a key role in modulating gut microbiota dynamics during experimental colitis. While overall alpha diversity remained stable, AnxA1^-/-^ mice exhibited earlier and more pronounced taxonomic shifts compared to WT, suggesting increased susceptibility to inflammation-associated dysbiosis. These changes, particularly involving reductions in SCFA-producing bacteria and expansions of *Proteobacteria*, likely reflect impaired regulation of immune–microbiota interactions in the absence of AnxA1. The findings indicate that AnxA1 contributes to gut homeostasis by fine-tuning microbial composition over time and orchestrating a more balanced response and recovery to inflammatory stress, highlighting its relevance in mucosal immunity and the resolution of inflammation.

To determine whether these findings translate to human disease, we further investigated the relationship between AnxA1 expression and microbiota composition in patients with IBD. Studies have shown that alpha-diversity in IBDs, particularly in CD, is typically reduced compared to healthy individuals, with lower species richness and uneven microbial distribution, often marked by a loss of beneficial bacteria. This decrease is associated with more severe symptoms and chronic inflammation (81). In this study, non-inflamed CD patients with low AnxA1 expression had reduced species richness compared to controls. Such differences were not seen in patients with higher AnxA1, regardless of inflammation. These findings suggest that AnxA1 may be associated with maintaining the balance of alpha-diversity in IBD, sustaining homeostasis and preventing the worsening of inflammation.

Although UC patients showed no difference in richness compared to controls, the evenness of bacteria in UC-inflamed subjects with high AnxA1 was decreased compared to controls. Thus, while bacterial distribution varied in this group, the genera and total number of bacteria still resembled those of a healthy control, suggesting that AnxA1, particularly its high expression, may contribute to maintaining eubiosis. Non-inflamed CD subjects with low expression of AnxA1 also presented less richness and evenness when compared to UC ones. This change may highlight how disease basal dynamics and location are also influenced by overall microbial composition and its interactions with the immune resolutive system.

In the present study, differences in the microbiota of inflamed patients with low AnxA1 expression in CD were found compared to controls, but not in individuals with high AnxA1 expression. No differences were found between inflamed individuals with high AnxA1 expression and controls. This shows that while healthy individuals and those inflamed with low AnxA1 expression have distinct microbiotas, individuals with high levels of AnxA1 display an intermediate profile between the two of them.

Moreover, UC patients with active inflammation but with low AnxA1 expression exhibited different microbial composition than their counterparts with higher AnxA1 levels. These differences were observed primarily at the phylum, class, and order levels, suggesting subtle but specific shifts in community structure even in the absence of overt inflammation. However, no significant differences were found between these UC subgroups and healthy controls, indicating that changes associated with reduced AnxA1 in UC may not yet reach the level of dysbiosis detectable against a healthy baseline. In contrast, CD patients without inflammation but with low AnxA1 expression showed a markedly different microbiota composition compared to UC subjects with similarly low AnxA1 expression. The most notable alterations also included an increase in *Lachnoclostridium* and *Parabacteroides* in CD, alongside a reduction in *Faecalibacterium*. These findings suggest that the impact of AnxA1 deficiency on microbiota composition may be disease-specific and more pronounced in the context of CD. Altogether, these results highlight a potential role for AnxA1 in shaping gut microbial ecology even in the absence of inflammation and raise the possibility that low AnxA1 expression may prime the intestinal environment for disease progression in a disease-dependent manner.

*Faecalibacterium* is a key butyrate-producing genus involved in maintaining intestinal homeostasis by promoting anti-inflammatory macrophage polarization (M2), inducing Treg differentiation, and stimulating IL-10 production (82). Its reduction in IBD has been associated with increased inflammation, impaired epithelial barrier function, and heightened innate immune activation (83, 84). Despite its known relevance, this study did not reveal a clear difference in *Faecalibacterium* abundance across stratified patient groups.

*Parabacteroides* is considered a context-dependent genus within the gut microbiota. Its role in the gut is not universally good or bad, but shaped by host–microbe interactions, microbial context, and immune status. In murine models, *Parabacteroides distasonis* has been shown to stimulate IL-10 production and attenuate experimental colitis (85, 86). Furthermore, a synergistic effect between *P. distasonis* and *Akkermansia muciniphila* has been reported to protect against both acute and chronic colitis by enhancing epithelial barrier integrity and promoting the accumulation of group 3 innate lymphoid cells (ILC3s) in the colonic mucosa (87). In CD, however, its colonization has been associated with depressive-like behaviors, possibly due to gut–brain axis interactions (88). The abundance of *Parabacteroides* is influenced by various factors, with some species displaying notable resilience to antimicrobial treatments (89, 90). In this study, inflamed CD patients with low AnxA1 expression exhibited approximately twice the abundance of *Parabacteroides* compared to controls and those with high AnxA1 levels, a pattern not observed in UC. In AnxA1^-/-^ mice, *Parabacteroides* levels increased throughout experimental colitis, with *Parabacteroides goldsteinii* being the only species identified. By day 10 of colitis, *P. goldsteinii* abundance was higher in AnxA1^-/-^ mice compared to WT. Overall, the genus remained more abundant in WT animals, suggesting complex and context-dependent regulatory dynamics mediated by AnxA1.

A key limitation of our study is that, although AnxA1 deficiency was associated with distinct microbial shifts and worsened colitis outcomes, our data do not allow us to determine whether these alterations in the microbiota act as causal drivers of pathology or merely represent secondary consequences of inflammation. Establishing this directionality would require dedicated mechanistic approaches, such as fecal microbiota transfer (FMT), to directly test whether the observed microbial profiles can modulate disease severity in the context of AnxA1 signaling. In this regard, our discussion of potential metabolic consequences was grounded in previously published functional studies of the bacterial taxa identified, providing a conceptual framework and rationale for future investigations to address this cause–consequence interplay.

Other resolvins, especially the ones derived from eicosapentaenoic acid-EPA RvE1 and RvE2, and from the docosahexaenoic acid-DHA RvD1, AT-RvD1 and RvD2, have emerged as pivotal regulators of intestinal inflammation (91). In experimental colitis models, they mitigate classical features of disease activity, including weight loss, colon shortening, neutrophil infiltration, and cytokine production, while simultaneously enhancing macrophage phagocytosis and epithelial barrier protection (92–94). Mechanistically, these resolvins signal through specific receptors, such as Leukotriene B4 receptor 1 (BLT1), Lipoxin A4 receptor (ALX)/FPR2, and Chemokine-like receptor 1 (CMKLR1), thereby dampening nuclear factor kappa B (NF-κB) activation, reducing adhesion molecule expression, and facilitating the non-inflammatory clearance of apoptotic cells (93, 95, 96).

In the clinical context, differential expression of resolvins has been linked to disease activity in both CD and UC. Notably, RvE1 levels were consistently lower in CD than in UC, and in the active phase, this lipid mediator emerged as a potential discriminator between the two entities. In UC patients, remission was also more strongly associated with RvE1 and correlated with lower clinical and endoscopic activity, suggesting its potential utility as a biomarker of mucosal healing (97, 98). Importantly, RvD2 also attenuates DSS-induced colitis, demonstrating efficacy comparable to anti-TNF therapy in preclinical settings, underscoring its translational potential (99).

Campbell et al. (100) demonstrated that RvE1 protects against DSS-induced colitis, likely through the activation of intestinal alkaline phosphatase, which helps detoxify bacterial lipopolysaccharides. Similarly, Zeng et al. (101) showed that RvD1 ameliorates hepatic steatosis in DSS-induced chronic colitis by reshaping the gut microbiota. RvD1 treatment restored a healthier microbial profile resembling that of control mice, normalized the *Firmicutes*-to-*Bacteroidetes* ratio, increased beneficial bacteria such as *Akkermansia*, and reduced genera like *Bacteroides* and *Desulfovibrio*. These microbiota changes were associated with improved intestinal barrier integrity and reduced systemic inflammation, suggesting that RvD1 exerts its protective effects through modulation of the gut-liver axis. Taken together, these observations highlight resolvins as not only candidate therapeutic targets but also as biomarkers capable of distinguishing disease phases and entities. Functionally, they appear to mirror several actions attributed to Annexin A1, supporting the notion that convergent pro-resolving pathways act in parallel to restrain intestinal inflammation and promote mucosal homeostasis.

In this context, studies investigating AnxA1 in the setting of IBD treatment have been increasingly reported. AnxA1-mimetic peptides, such as Ac2-26, reproduce many of the anti-inflammatory and pro-resolving actions of the native protein, including inhibition of neutrophil infiltration, promotion of macrophage-mediated clearance, and restoration of epithelial barrier integrity (38). Polymeric nanoparticles loaded with Ac2–26 have been tested in murine models of colitis, where they accelerated epithelial wound healing and promoted intestinal tissue regeneration (40). Li et al. (102) further demonstrated that Ac2-26-containing nanoparticles reshaped the microbiota of DSS-induced mice, reducing the expansion of *Escherichia–Shigella* and increasing levels of *Prevotellaceae*. Alpha and beta diversity did not differ from control groups after nanotherapy treatment. Taken together with our findings, and supported by previous evidence showing that AnxA1 deficiency alters gut microbial composition and exacerbates colitis, these peptides may represent a dual-action therapeutic strategy: directly resolving inflammation while indirectly modulating the gut microbiota. Future studies are warranted to determine whether AnxA1-mimetic peptides can favorably reshape microbial communities and translate these mechanistic insights into therapeutic benefit for patients with IBD.

Altogether, the results presented here suggest that AnxA1 plays a key role in shaping the intestinal microbiota through its immunomodulatory effects, both in experimental colitis and in IBD patients. In murine models, the absence of AnxA1 led to early and more pronounced dysbiotic shifts during colitis. Even under healthy conditions, AnxA1^-/-^ mice exhibited reduced microbial richness and distinct community structures, indicating that AnxA1 contributes to maintaining microbiota resilience and mucosal homeostasis. In human cohorts, patients with higher AnxA1 expression, regardless of inflammatory status, had microbial profiles more similar to healthy controls, with greater uniformity and richness, suggesting that AnxA1 may protect against microbiota disruption and excessive inflammation. Differences in microbial composition among IBD subtypes further suggest that the influence of AnxA1 on the gut ecosystem may be disease-specific and dependent on the inflammatory context. Hence, these findings underscore the critical role of AnxA1 in orchestrating host–microbiota interactions and maintaining intestinal equilibrium. They also highlight the therapeutic potential of targeting AnxA1 pathways to restore microbiota balance and reduce inflammation in IBD.

## Data Availability

The datasets analyzed for this study can be found in the European Genome-Phenome Archive https://ega-archive.org/studies/EGAS00001005027.
