# Supplementary figures and images for: Annexin A1 levels affect microbiota in health and DSS-induced colitis/inflammatory bowel disease development
Source: Front Immunol. 2025 Oct 3;16:1679071. doi: 10.3389/fimmu.2025.1679071 (PMC12531217; doi:10.3389/fimmu.2025.1679071)

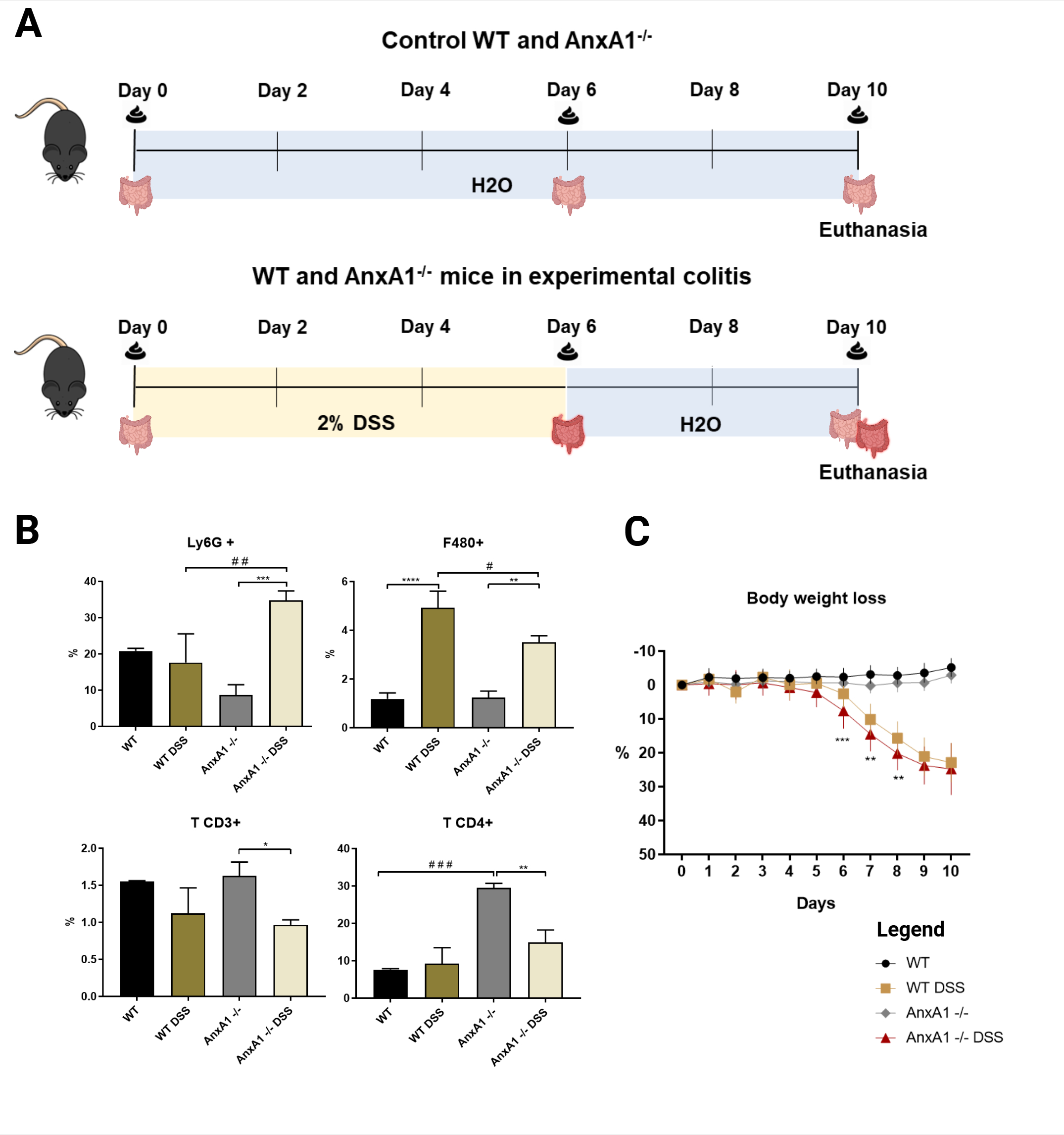

Supplement: Supplementary file 1 [file Image1.jpeg]
